# Frameworks for cultural adaptation of psychosocial interventions: A systematic review with narrative synthesis

**DOI:** 10.1177/14713012231192360

**Published:** 2023-07-28

**Authors:** Sally Day, Kate Laver, Yun-Hee Jeon, Kylie Radford, Lee-Fay Low

**Affiliations:** Faculty of Medicine and Health, 522555 The University of Sydney, Sydney, NSW, Australia; College of Medicine and Public Health, 64767 Flinders University, Adelaide, SA, Australia; Faculty of Medicine and Health, 522555 The University of Sydney, Sydney, NSW, Australia; 6803 Neuroscience Research Australia, Randwick, NSW, Australia; School of Psychology, 441985 University of New South Wales, Sydney, NSW, Australia; Ageing Futures Institute, 567274 University of New South Wales, Sydney, NSW, Australia; Faculty of Medicine and Health, 522555 The University of Sydney, Sydney, NSW, Australia

**Keywords:** systematic review, cultural adaptation, frameworks, psychosocial intervention, adaptation, culture, dementia

## Abstract

**Introduction:**

Psychosocial dementia interventions may be less effective when used with populations for whom they were not initially intended. Cultural adaptation of interventions aims to increase effectiveness of interventions by enhancing cultural relevance. Use of theoretical frameworks may promote more systematic cultural adaptation. The aim of this review was to provide a comprehensive synthesis of published cultural adaptation frameworks for psychosocial interventions to understand important elements of cultural adaptation and guide framework selection.

**Method:**

Five scientific databases, grey literature and reference lists were searched to January 2023 to identify cultural adaptation frameworks for psychosocial interventions. Papers were included that presented cultural adaptation frameworks for psychosocial interventions. Data were mapped to the framework for reporting adaptations and modifications to evidence-based interventions, then analysed using thematic synthesis.

**Results:**

Twelve cultural adaptation frameworks met inclusion criteria. They were mostly developed in the United States and for adaptation of psychological interventions. The main elements of cultural adaptation for psychosocial interventions were modifying intervention content, changing context (where, by whom an intervention is delivered) and consideration of fidelity to the original intervention. Most frameworks suggested that key intervention components must be retained to ensure fidelity, however guidance was not provided on how to identify or retain these key components. Engagement (ways to reach and involve recipients) and cultural competence of therapists were found to be important elements for cultural adaptation.

**Conclusions:**

Comprehensive frameworks are available to guide cultural adaptation of psychosocial dementia interventions. More work is required to articulate how to ensure fidelity during adaptation, including how to identify and retain key intervention components.

## Introduction

Psychosocial interventions can improve quality of life, delay functional decline, and reduce depression in people with dementia and their families (Bennett et al., 2019; Laver et al., 2016; Watt et al., 2021). Psychosocial interventions may involve use of psychological, behavioural and/or social approaches or strategies to improve individuals’ outcomes (England et al., 2015). However, a growing body of work has argued that interventions developed for one population may not be effective when directly applied to other cultures or populations (e.g., Cardemil, 2010; Castro et al., 2010; La Roche & Christopher, 2009; Lau, 2006). Developing a new intervention for each new population can be costly and time consuming (Castro et al., 2010).

One approach to address this issue is cultural adaptation, “*the systematic modification of an evidence-based treatment (EBT) or intervention protocol to consider language, culture, and context in such a way that it is compatible with the client’s cultural patterns, meanings, and values”* (Bernal et al., 2009, p. 362). Cultural adaptation of dementia interventions is increasingly conducted (e.g., Brijnath et al., 2022; Parker et al., 2022) and in particular should be considered when an intervention is found to be less effective with a new population (Cardemil, 2010). New populations may have different characteristics compared with the original intervention population or their response to the intervention may differ (Lau, 2006).

Cultural adaptation changes cultural sensitivity including both “surface level” changes (e.g., matched language and/or images in intervention materials) to increase acceptability and “deep structural” changes (e.g., incorporation of cultural values) to ensure intervention impact (Resnicow et al., 1999). For example, a skills training intervention for caregivers of people with dementia was adapted for Latino populations in the United States (US) to include resources in Spanish and English, bicultural workers to deliver the intervention, and increased use of visual aids and verbal activities to account for lower literacy levels (Gallagher-Thompson et al., 2003). Culturally adapted psychosocial interventions, including those for people with dementia, have been shown to be effective for minority groups in Western countries, and people from non-Western and lower-middle income countries (LMICs) (e.g., Arundell et al., 2021; Hall et al., 2016; James et al., 2021; Rathod et al., 2018)

The many challenges of cultural adaptation have been extensively documented and continue to be explored (Barrera & Castro, 2019; Cardemil, 2010, 2015, 2015; Castro et al., 2010; Miranda et al., 2005). Foremost is the “Fidelity-Adaptation Dilemma” (Castro & Yasui, 2017), describing the tension between delivery of evidence-based interventions as developed to ensure effectiveness and the need to address local population needs by making changes. Cultural adaptation specifically considers where there is intervention-population mismatch and makes purposeful changes that aim to increase the relevance and fit (Castro et al., 2010). However, this presents a risk that the intervention may be adapted in a way that decreases effectiveness (Cardemil, 2010). It has been suggested that fidelity can be maintained by strategic cultural adaptation that retains an intervention’s core theoretical components while making changes to ensure cultural relevance for the new population (Castro & Yasui, 2017). There are some examples of this with psychosocial interventions for people with dementia and their families; James and colleagues (2021) showed that evidence-based dementia interventions that retained the core therapeutic components of the intervention remained effective when adapted for local contexts, such as incorporating local language and customs, removal of reading and writing tasks or adding services such as blood pressure screening to increase attendance.

Researchers and practitioners can apply cultural adaptation frameworks to support transparent, replicable adaptation of dementia psychosocial interventions; these are defined as *“a systematic way to carry out and document adaptations that can be useful for planning, replication, dissemination and translation”* (Bernal et al., 2009, p. 364). Frameworks may be process models (i.e., directed steps for adaptation) or content-specific models (i.e., consideration of areas of the intervention that can be targeted for adaptation) or a combination of both (Ferrer-Wreder et al., 2012). Frameworks are designed to provide consistency and clarity in cultural adaptation research and to assist with real-world implementation.

As cultural adaptation research has increased over the past 20 years (Rathod et al., 2018), there has also been an increase in published cultural adaptation frameworks. Practitioners and researchers need guidance to select the appropriate framework for their intervention and populations (Rathod et al., 2018). Two recent reviews summarise available guidelines and frameworks for adaptation of health interventions (Escoffery et al., 2019; Movsisyan et al., 2019). Both reviews emphasised the important steps for adaptation: planning (gather information, assess the community, select the intervention, consult with stakeholders); conducting (decide what needs to be adapted then adapt the intervention) and testing (pilot, implement, evaluate). The engagement of the local community was identified as important for meaningful adaptation. From these reviews, the steps to guide adaptation are clear (i.e., planning, conducting, testing); they are included in “process model” cultural adaptation frameworks. It is less clear which aspects of the intervention should be considered for adaptation. Both reviews included cultural adaptation frameworks, but an in-depth exploration was out of scope.

The aim of this review was to provide a comprehensive synthesis of published cultural adaptation content-specific frameworks.

Specific questions were:

• What frameworks for cultural adaptation of psychosocial interventions are publicly available and how have they been applied?
• What are the main elements of cultural adaptation included in the identified frameworks?
• What are the commonalities and differences across the frameworks?

## Methods

The systematic review protocol was registered prospectively on the PROSPERO database (CRD42021266156). Reporting is according to the Preferred Reporting Items for Systematic Reviews and Meta-Analyses (PRISMA) 2020 guidelines (Page et al., 2021) (see APPENDIX for PRISMA checklist).

### Eligibility criteria

Papers were included that discussed a new content-specific framework for cultural adaptation of face to face, psychosocial interventions. The search was not limited to empirical studies and included all paper types (systematic reviews, primary research papers, commentaries, etc.)

Included articles considered all of the following:

1) Cultural adaptation: the “the systematic modification of an evidence-based treatment (EBT) or intervention protocol to consider language, culture, and context in such a way that it is compatible with the client’s cultural patterns, meanings, and values” (Bernal et al., 2009, p. 362)
2) Psychosocial interventions: interventions with a focus on non-pharmacological approaches to improving (or reducing risk to) psychological, social and everyday functional abilities (England et al., 2015)
3) Frameworks: graphical or narrative representations of the cultural adaptation process (Ferrer-Wreder et al., 2012)
  a. Specifically, content-specific frameworks which target areas of an intervention that may need to be considered for adaptation (Ferrer-Wreder et al., 2012)

Articles were excluded if they focused on any of the following:

1) Theory of cultural adaptation, with no specified framework (e.g. Bernal et al., 2009; Castro et al., 2004; Domenech Rodriguez & Bernal, 2012; Ferrer-Wreder et al., 2012; Lau, 2006)
2) “Process model” frameworks that describe a series of pre-defined steps to conduct the adaptation (e.g., Aguirre et al., 2014; Fendt-Newlin et al., 2020; Hwang, 2009; Kumpfer et al., 2008; McKleroy et al., 2006; Perera et al., 2020; Wingood and DiClemente, 2008)
3) Application or validation of an existing framework without proposing any changes to the original framework (e.g., Naeem et al., 2019; Rathod et al., 2019)
4) Frameworks to develop culturally specific interventions, not for adapting an existing intervention (e.g., Hockley et al., 2019)
5) Validation or adaptation of standardised assessment tools rather than an intervention; and/or
6) Frameworks about adaptation of psychosocial interventions relating to technology or technological interventions (i.e., not face to face)

### Information sources and search strategy

Five health and social care academic databases (PsycINFO, Embase, Medline, CINAHL and Scopus) were searched up to 13 January 2023. SEE ADDITIONAL FILE Table 1. A search of Google Scholar (first 400 references) was also conducted (Bramer et al., 2017). The search strategy was developed in conjunction with an experienced Health Sciences Librarian and mapped to medical subject headings (MeSH) and text words in MEDLINE. This strategy was adapted for the other databases. The strategy combined terms related to cultural adaptation AND frameworks AND psychosocial intervention (see Table 1 for search terms). No date limits were imposed on the search because we were interested in the development and evolution of frameworks over time. Full Medline search string is provided in ADDITIONAL MATERIAL Table 2. References were imported to Covidence, a systematic review management system (Covidence systematic review software, 2021). Reference lists of all included papers were hand searched for additional relevant papers.

**Table 1.** Concepts and search terms.

Concepts	Search terms
Cultural adaptation	cultur* adapt* OR cross?cultur* OR cultural* tailor* OR cultural* safe* OR cultural* responsive* OR cultur* adjust* OR cultur* modif*
Frameworks	frame?work* OR guideline* OR model* OR theor*
Psychosocial intervention	psychosocial intervention OR psychoeducation OR social intervention OR function* intervention OR behavio?r* intervention* OR skills training OR cognitive behavio?ral therap* OR occupational therap* OR cogniti* rehabilitation OR psycholog* intervention OR psychotherap*

*Note.* All three concepts need to be included: cultural adaptation AND frameworks AND psychosocial intervention.

### Selection process

Search results were exported into Covidence and duplicates were removed. Two reviewers (SD and either LFL or YHJ) independently screened titles and abstracts for eligibility. Abstracts not available in English were translated using google translation software. Inconsistencies were discussed until consensus reached. In case of disagreement, articles were included for full-text review. Full texts were reviewed against eligibility criteria by at two reviewers (SD and LFL). Again, consensus was reached on inclusion or exclusion.

### Data extraction

Data were extracted by SD using a specifically designed data extraction sheet that was tested with one study, reviewed by SD and LFL and revised. Data extraction included country, framework ‘demographics’ (name, description, target population and intervention), verbatim framework description, including visual representation (where available), theoretical underpinning, methodology of framework development and key concepts surrounding framework (definitions used, rationale for adaptation). A summary of extracted data is presented in Table 2. Once completed the data extraction was shared with all authors to ensure the information extracted was appropriate and adequate. As this review focused on the frameworks presented rather than the robustness of the studies, a quality assessment of the articles was not conducted.

**Table 2.** Framework characteristics.

First author, year, country	Framework name	Intervention area	Target group	Development methodology	Intended usage	Theoretical foundations	Description
Barrera & Castro, 2006, USA	A Heuristic Framework for the cultural adaptation of interventions	Psychology	Various^a^	Theoretical model	Research guide to plan and evaluate adaptations of existing interventions	- Selected and directed approach to cultural adaptation (Lau, 2006)	-Conceptual framework representation of Lau’s (2006) work
						- Re-AIM (Glasgow, 1999) engagement factors	-Incorporates specific target areas for adaptation in the areas of engagement and outcomes
						- Small theory (Lipsey, 1993) outcomes	
						- Action theory (Chen, 1990)	
Bernal et al., 1995, USA	Ecological Validity Framework (EVF)	Psychotherapy	Latinos	Literature review	Guide to adapt existing interventions	- Ecological validity (Bronfenbrenner, 1977)	-Matches eight dimensions of interventions and corresponding culturally sensitive elements for adaptation
						- Cultural sensitivity (Malgady et al., 1990; Rogler, 1989)	- Dimensions: Language, persons, metaphors, content, concepts, goals, methods and context
Chu & Leino, 2017, USA	Cultural Treatment Adaptation Framework (CTAF)	Mental health interventions	Various^a^	Literature review	Train providers;	None (specifically states other frameworks have been based in theory and this one is grounded in actual data from cultural adaptation research)	Two main categories to consider for adaptation
					Replicate and validate adapted interventions		• Peripheral components, encompassing engagement and treatment delivery: “*increase how receptive or understandable a treatment is to a particular population*”
							• Core components: “*primary therapeutic elements responsible for mediating symptom change*”
Heim & Kohrt, 2019, Switzerland	New framework for cultural adaptation	Psychological interventions	Various^a^	Theoretically developed	Guide empirical research on how much and where to invest in cultural adaptation of psychological interventions	- Ethnopsychology (White, 1992)	Three elements for adaptation
						- Surface and deep structure adaptation (Resnicow et al., 1999)	• Cultural concepts of distress
						- Psychotherapy research, namely whether the effect of the treatment is rooted in the techniques themselves or the rationale for their use (Wampold and Imel, 2015)	• Treatment components (specific, non-specific and in-session techniques), and
							• Treatment delivery: *“surface adaptations to engage with cultural concepts of distress and deliver treatment options”*
							All elements can be modified to cause symptom change
Hwang, 2006, USA	Psychotherapy Adaptation and Modification Framework (PAMF)	Psychotherapy	Various^a^ (applied to Asian Americans)	Literature review	Support clinical researchers to adapt evidence-based interventions	- Cultural influences on mental health model (Hwang et al., 2006)	- Top down, theoretically driven approach to cultural adaptation
				+ clinical expert consultation	Train practitioners in cultural competence	- Therapeutic principles for understanding and treating Chinese Americans (Hwang et al., 2008)	- Descriptive framework
				+ clinical experience	Facilitate clinical training by educators		- Maps six therapeutic domains to 25 therapeutic principles providing rationale for cultural adaptations
Leong & Lee, 2006, USA	Cultural Accommodation Model (CAM)	Psychotherapy	Various^a^ (applied to Asian Americans)	Theoretically developed	Tool for therapists in culturally diverse therapeutic relationships	- Integrative model of cross-cultural psychology (Leong & Lee, 2006; Leong, 1996)	Three phases to of cultural accommodation
							• Identify cultural gaps that restrict cultural validity
						- Cultural validity of psychological constructs (Leong & Brown 1995)	• Select culturally specific concepts to fill gap
							• Test the culturally accommodated theory
Marinez-Lora & Atkins, 2012, USA	Combined cultural adaptation model for Latinos	Behavioral parent training	Latinos	Combination of existing models	Inform research and clinical practice about quality of cultural adaptations;	- Selected and directed approach to cultural adaptation (Lau, 2006)	Combines the dimensions of the with the adaptation analysis provided by Lau
					Provide guidelines for effective adaptations	- Ecological validity Framework (Bernal et al., 1995)	
Naeem et al., 2016, Canada/UK	Framework for cultural adaptation of cognitive behaviour therapy	Cognitive behavior therapy	Non-Western cultures	Literature review + clinical expert consultation	Adapt CBT for clients from non-Western cultural backgrounds	Not reported	Four overarching concepts
							• Philosophical orientation, technical adjustments required, practical considerations of adaption and theoretical modifications, underpinned by three delivery areas:
							• Awareness, assessment and engagement and adjustments in therapy
Patchell et al., 2012, USA	Circular model of cultural tailoring	Prevention programs	Native Americans	Clinical experience	Develop or implement culturally driven interventions	- Cherokee Self Reliance model (lowe, 2002)	Four multi-level elements
						- Cultural tailoring (Domenech Rodriguez et al., 2011)	• self, time, relationships and tribe which support
							Three patterns of knowing
							• Expression of culture, lived experience of culture and a central flashpoint which together provide a circular framework for cultural adaptation.
							Elements are positioned as points on a compass, or Native American medicine wheel, providing further depth to the cultural adaptation
Sangraula et al., 2021, Nepal	mental health Cultural Adaptation and Contextualization for Implementation (mhCACI)	Psychological interventions	LMIC^b^	Combination of existing models	Prepare psychological interventions for widespread implementation in an LMIC or humanitarian setting	- Ecological validity Framework (Bernal et al., 1995)	Step-wise model that incorporates specific concepts for adaptation from the EVF at each step
						- Replicating effective programs framework (Kilbourne et al., 2007)	
Sorenson & Harrell, 2021, USA	4-Domain Cultural Adaptation Model (CAM4)	Psychological interventions	Various^a^	Literature review	For professionals to acquire cultural knowledge	- Adaptation theory (McCrae and Purssell, 2018)	Four domains for consideration in the adaptation process
					Provide roadmap for what kind of knowledge to seek	- Cultural competence (Sue et al., 1998)	• Development and equivalence
					Guide the tailoring of evidence-based interventions	- Ecological validity Framework (Bernal et al., 1995)	• Cultural content and context
						- Psychotherapy adaptation and modification Framework (Hwang, 2006)	• engagement
						- Selected and directed approach to cultural adaptation (Lau, 2006)	• Cultural competence
						- A Heuristic Framework for the cultural adaptation of interventions (Barrera & Castro, 2006)	
						- Cultural treatment adaptation Framework (Chu & Leino, 2017)	
						- Cultural adaptation theoretical and empirical studies (multiple authors)	
Whitbeck, 2006, USA	Theoretical model for developing culturally specific preventions with Native American people	Prevention programs	Native Americans	Clinical experience	Culturally adapt key constructs to fit the social context;	Ethical guidelines for researchers working with Native Americans	Combines steps for adaptation with guiding assumptions for research partnerships
					Highlight the effects of culturally specific risk and protective factors		

^a^Ethnically and culturally diverse groups.

^b^Low Middle-Income Countries.

### Narrative synthesis

A narrative approach to data synthesis was utilised: 1) conducting a preliminary synthesis, 2) conducting thematic analysis, 3) exploring concepts across papers, and 4) assessing the robustness of the synthesis (Popay et al., 2006).

#### Preliminary synthesis

The Framework for Reporting Adaptations and Modifications – Enhanced (FRAME) (Wiltsey Stirman et al., 2019) was used as the basis for preliminary tabulation of common adaptation elements identified in the cultural adaptation frameworks. FRAME was developed to support the systematic reporting of modifications to evidence-based interventions during intervention adaptation or implementation (Stirman et al., 2013; Wiltsey Stirman et al., 2019). It describes the different adaptation features: reasons for, the process, including what is modified and the nature of the modifications, and fidelity to the original intervention. SD initially tabulated the adaptation elements from each framework to FRAME, all authors independently reviewed the tabulation, and any disagreements were discussed as a whole group.

#### Thematic synthesis

Extracted data that did not map onto FRAME during preliminary synthesis were examined using thematic synthesis (Thomas & Harden, 2008). Each line of data extracted was coded by SD and themes developed in collaboration with all authors using a deductive approach to analysis based on cultural adaptation theory (e.g., Bernal et al., 2009; Cardemil, 2010; Lau, 2006). Coded data was cross-checked with original papers for context within each framework by SD and final accuracy of interpretation was discussed and agreed by all authors. Finally, two additional common cultural adaptation elements were identified; engagement and cultural competence (see Box 1 for definitions).

### Box 1:

#### Definitions of adaptation elements

**FRAME elements:**

• **Content:** “changes made to the intervention procedures, materials or delivery” p. 7 ^1^
• **Context:** “changes made to delivery of the same program content, but with modifications to the format or channel, the setting or location in which the overall intervention is delivered, or the personnel who deliver the intervention….[and] the population to which an intervention is delivered” p.6 ^1^
• **Training and Evaluation:** “changes made ‘behind the scenes’ during an implementation effort” p.4 ^1^
• **Implementation and scale up activities:** “If little about the intervention were changed, but the implementation strategies differed significantly across otherwise similar contexts, then differences in outcomes may be attributable to (these) differences”. p.5 ^2^
• **Intervention fidelity:** “Fidelity-consistent modifications are defined as those that preserve core elements of a treatment that are needed for the intervention to be effective” p.5 ^2^

**Additional elements identified through thematic analysis:**

• **Engagement:** “the ability of procedures to reach potential participants and involve them successfully in the intervention” p.311 ^3^
• **Cultural competence:** “a set of congruent behaviors, attitudes, and policies that come together in a system, agency, or amongst professionals and enables that system, agency, or those professionals to work effectively in cross-cultural situations” p.13 ^4^

1: Stirman et al., 2013, p. 2: Wiltsey Stirman et al., 2019, p. 3: Barrera & Castro, 2006, p. 4: Cross et al., 1989

#### Exploring concepts

Commonalities and differences between frameworks were explored by SD graphing the cultural adaptation elements identified in steps 1 and 2 (Popay et al., 2006) and all authors further analysing the data through thematic synthesis by developing analytic themes and grouping themes across frameworks (Thomas & Harden, 2008).

#### Assessing robustness of synthesis

Robustness of the synthesis was achieved by all authors examining, discussing and refining the analysis at each stage: preliminary synthesis, thematic synthesis, the identified adaptation elements, descriptions of each element and the synthesised analytic themes. Analysis was revised and finalised following critical reflection.

## Results

**Figure 1.**
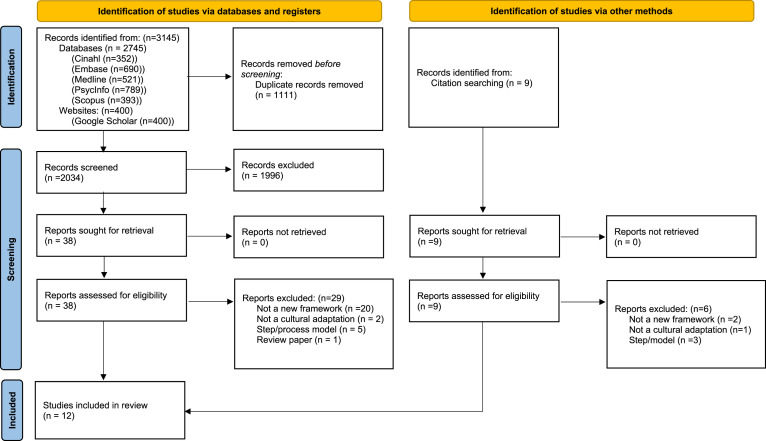
PRISMA flow diagram.

Of the 2034 unique records identified through the database search (after duplicates removed), most were excluded (*n* = 1996) based on the title and abstract (see Figure 1). Thirty-eight full text articles were reviewed. Nine met the inclusion criteria (Barrera & Castro, 2006; Bernal et al., 1995; Chu & Leino, 2017; Heim & Kohrt, 2019; Hwang, 2006; Marinez-Lora & Atkins, 2012; Patchell et al., 2012; Sangraula et al., 2021; Sorenson & Harrell, 2021). Three additional eligible papers were found from hand searching reference lists of included papers (Leong & Lee, 2006; Naeem et al., 2016; Whitbeck, 2006). A total of 12 papers are included in this review.

### Characteristics and purpose of cultural adaptation frameworks

Included papers were published between 1995 and 2021. Most were from the USA (*n* = 9) (see Table 2). Most frameworks were for adaptation of non-specific psychological or mental health interventions (*n* = 8), others were for prevention programs (*n* = 2), behavioural parent training (*n* = 1) and one specifically for the adaptation of Cognitive Behavioural Therapy (CBT). No frameworks were specifically designed for dementia interventions. Six frameworks were developed for adaptation for any ethnic or cultural minority groups in a Western country, two frameworks were for adaptation for non-Western cultures or LMICs and four were for specific cultural groups in the USA (Latinos, *n* = 2; Native Americans, *n* = 2).

Most frameworks (*n* = 9) were primarily for application in guiding practitioners and local stakeholders through cultural adaptations (Bernal et al., 1995; Hwang, 2006; Leong & Lee, 2006; Marinez-Lora & Atkins, 2012; Naeem et al., 2016; Patchell et al., 2012; Sangraula et al., 2021; Sorenson & Harrell, 2021; Whitbeck, 2006). The other frameworks were applied in research: to test cultural adaptations (Barrera & Castro, 2006), validate adaptations (Chu & Leino, 2017) and to inform future empirical research (Heim & Kohrt, 2019). Three frameworks were also intended for clinical training (Chu & Leino, 2017; Hwang, 2006; Marinez-Lora & Atkins, 2012).

### Commonalities and differences across frameworks

#### Theoretical foundations:

Frameworks had diverse theoretical foundations, most incorporating multiple theories (see Table 2). Some frameworks (*n* = 4) were derived from cultural adaptation theory (Barrera & Castro, 2006; Heim & Kohrt, 2019; Marinez-Lora & Atkins, 2012; Sorenson & Harrell, 2021), others (*n* = 3) from cultural sensitivity literature for a specific cultural group (Hwang, 2006; Patchell et al., 2012; Whitbeck, 2006) and one from cultural validity of interventions (Leong & Lee, 2006). Bernal et al. (1995) developed the Ecological Validity Framework (EVF) based on the premise that ecological validity is strengthened through cultural sensitivity of interventions; some frameworks (*n* = 3) drew on the EVF as their theoretical foundation (Marinez-Lora & Atkins, 2012; Sangraula et al., 2021; Sorenson & Harrell, 2021). The mhCACI (Sangraula et al., 2021) specifically drew on implementation theory. The varied theoretical foundations and development methodologies were reflected in how the cultural adaptation elements were defined and used, and the emphasis during the cultural adaptation process.

#### Elements considered during cultural adaptation:

Content-specific frameworks refer to any areas of an intervention that may need to be factored into adaptation (Ferrer-Wreder et al., 2012). The identified cultural adaptation elements from the framework synthesis were context, content, fidelity, engagement and cultural competence. Included frameworks varied in their inclusion of the considered adaptation elements (see Table 3). Although included in the preliminary synthesis, less than half the frameworks mentioned ‘Training and evaluation’ (*n* = 5) and ‘Implementation and scale-up activities’ (*n* = 2). These adaptation elements reflect the background work required to support implementation (Stirman et al., 2013). While differences in implementation may impact on intervention effectiveness (Stirman et al., 2013), these elements did not have cultural adaptation as the focus and therefore were not analysed further. The ‘4-Domain Cultural Adaptation Model (CAM4)’ was the only framework to encompass all the cultural adaptation elements.

**Table 3.** Summary of included cultural adaptation elements.

		Heuristic framework^1^	Ecological validity framework^2^	Cultural treatment adaptation framework^3^	New framework for cultural adaptation^4^	Psychotherapy adaptation and modification framework^5^	Cultural accommodation model^6^	Combined cultural adaptation model^7^	Framework for cultural adaption of CBT^8^	Circular model of cultural tailoring^9^	Mental health cultural adaptation and contextualization for implementation^10^	4-Domain cultural adaptation model^11^	Developing culturally specific preventions for native americans^12^
Does the framework address?													
FRAME adaptation elements	Content (Content adaptation sub-elements)	+	+	+	+	+	+	+	+	+	+	+	+
	Tailoring/Tweaking/Refining	+	+	+	+	+	+	+	+	+		+	+
	Changes in packaging or materials		+	+	+			+	+		+	+	
	Changing elements	+	+	+	+		+	+	+	+			+
	Pacing/Timing			+						+			
	Integrating another approach into the intervention		+			+	+	+		+	+	+	+
	Integrating the intervention into another approach									+			
	Context (Context adaptation sub-elements)	+	+	+	+	+	+	+	+	+	+	+	+

	Format		+	+	+	+		+					
	Setting		+	+				+	+		+		
	Personnel		+	+							+		
	Population		+			+	+	+	+	+	+	+	+
	Training and evaluation	+	+				+				+		+
	Implementation and scale up activities	+									+		
	Intervention fidelity/role of core intervention components	+	+	+	+			+			+	+	+
Additional elements of cultural adaptation													
Engagement		+	+	+	+	+	+		+	+		+	
Cultural competence						+	+		+	+		+	+

1 = Barrera(2006), 2 = Bernal(1995), 3 = Chu(2017), 4 = Heim(2019), 5 = Hwang(2006), 6 = Leong(2006), 7 = Marinez-Lora(2012), 8 = Naeem(2016), 9 = Patchell(2012), 10 = Sangraula(2021), 11 = Sorenson(2021), 12 = Whitbeck(2006).

### Analysis of cultural adaptation elements:

#### Content

All twelve frameworks suggested cultural adaptation includes modification of the content of the original intervention. Methods of content modification varied (see Table 4). Eleven frameworks specified tailoring of intervention content such that intervention concepts and delivery were culturally sensitive and relevant to the new target population, such as including culturally representative images. Frameworks also specified adding or removing specific content (*n* = 9 frameworks), for example including immigration stress as a risk factor (Barrera & Castro, 2006) or traditional practices as a protective factor for the new cultural group (Whitbeck, 2006). Content also could be adapted to incorporate other cultural knowledge, and Indigenous or traditional beliefs and practices (*n* = 8 frameworks), for instance accommodation of individualism versus collectivism (Leong & Lee, 2006). Of these, only five frameworks specifically considered traditional or Indigenous knowledge (Hwang, 2006; Marinez-Lora & Atkins, 2012; Patchell et al., 2012; Sorenson & Harrell, 2021; Whitbeck, 2006). These frameworks guide culturally sensitive adaptation through partnership with local communities and incorporation of traditional knowledge and values.

**Table 4.** Synthesis of adaptation elements.

Adaptation elements	Type of adaptation	How elements are adapted
Content		
	Tailoring/Tweaking/Refining	Change or target intervention content where original is poor fit with new target population^3,4^
		Align explanation of intervention principles with new culture^2,4-6,8^
		Understand and incorporate culturally specific concepts of illness^4,5,8,11^
		Adjust therapeutic style, treatment methods and delivery, e.g. orient clients to intervention; know when to generalize and when to individualise^1,3-5,8,11^
		Frame intervention according to characteristics (experiences, beliefs, values and culture) of the new population^2,3,5,7,8,9,11,12^
		Ensure new population feel ownership of adapted intervention^9,12^
	Changes in packaging or materials	Translate materials^10^
		Culturally match images^3,10,11^
		Use meaningful and culturally appropriate language, symbols and communication^2-4,7,8,11^
		Include cultural stories and themes^2,3^
	Adding or removing elements	Add or remove content where new target population has unique risk or protective factors affecting how program components work^1,3,7,12^
		Evaluate cultural uniqueness, cultural values and add cultural elements to intervention components^2,6,7,9^
		Introduce culturally sensitive specific techniques or assessments^2,4,8,12^
	Pacing/Timing	Change format (e.g. time spent on each part of session or length of session) or number of sessions^3,9^
	Integrating another approach into the intervention	Consider pre-existing site practices^10^
		Include cultural knowledge (generally)^2,6,7^
		Incorporate traditional/Indigenous knowledge, beliefs and healing practices^5,9,11,12^
	Integrating the intervention into another approach	Start with Indigenous/traditional framework and embed intervention components/strategies^9^
Context		
	Format	Offer program flexibility^2,4,7^
		Program delivery may need to vary (e.g. group v individual)^3^
		Collaborate with family^5^
	Setting	Consider acceptability, inclusivity and practicality of location for new target group^2,3,7,8^
		Deliver specialist intervention within routine healthcare^10^
	Personnel	Reflect on the impact of client and therapist ethnic/racial match (or not)^2,7^
		Deliver by generic health care workers instead of specialist staff^10^
		Ensure workers are trained in both the intervention and how it works^10^
		Include other stakeholders in program delivery^3^
	Population	Consider how the new population or group may differ to original intervention population
		LMIC/low resource setting^10^
		Impact of life experiences: e.g. acculturation, migration stress, racism, cultural/family expectations^2,5-9^
		Unique cultural factors
		- Cultural beliefs^6,8^
		- Role of family, community and elders^8,9^
		- Conflict between therapeutic approach and cultural beliefs (e.g. health beliefs, stigma, help-seeking behaviour)^5,8^
		- Desire for cultural knowledge^12^
		Experiences
		- Socio-political, economic contextual considerations^2,5,7,11^
		- Unique local factors^11,12^
Relationship fidelity/Core elements		Align intervention components with cultural views to enhance effectiveness^2,12^
		Consider that the way intervention components lead to therapeutic outcomes may vary for diverse groups^1,4^
		Adhere to program fidelity while making adaptations^7^
		Retain key active components to ensure fidelity^3,10,11^
Engagement		
	Awareness	Awareness of intervention^1^
		Feasibility of intervention for new population/group^3^
	Access/Entry	Address facilitators & barriers to services/program^1,3,6,8,11^
		Acknowledge stigma (of therapy/intervention and/or illness)^5,11^
		Understand help seeking behaviour/pathways to care^5,8^
		Support understanding of/orientation to intervention^5,8,11^
	Client/Therapist relationship	Create therapeutic relationships^2,3,5,8,9^
		Recognize pre-existing beliefs about and expectations of therapists^5,8,9^
		Attain therapist credibility^2,5,9^
	Retention/Completion	Retention in/Completion of intervention^1,3,8^
Cultural competence		Be aware and knowledgeable of cultural ways^8,11,12^
		Express cultural humility and self-awareness of own values^5,8,9,11^
		Adjust therapeutic approach^5,6,8,11^
		Appreciate cultural factors relevant to each individual^5,6^

1 = Barrera(2006), 2 = Bernal(1995), 3 = Chu(2017), 4 = Heim(2019), 5 = Hwang(2006), 6 = Leong(2006), 7 = Marinez-Lora(2012), 8 = Naeem(2016), 9 = Patchell(2012), 10 = Sangraula(2021), 11 = Sorenson(2021), 12 = Whitbeck(2006).

#### Context

Most frameworks (*n* = 11) suggested adaptation of context (population, setting, format and personnel) (Bernal et al., 1995; Chu & Leino, 2017; Heim & Kohrt, 2019; Hwang, 2006; Leong & Lee, 2006; Marinez-Lora & Atkins, 2012; Naeem et al., 2016; Patchell et al., 2012; Sangraula et al., 2021; Sorenson & Harrell, 2021; Whitbeck, 2006). However, consideration of the context for cultural adaptation differed across frameworks. One example is how they considered adaptation to accommodate the differences between the new population and the original intervention group (identified in *n* = 9 frameworks). Some frameworks incorporated consideration of unique cultural factors or individual and societal experiences, such as experiencing racism (e.g., Naeem et al., 2016), whereas the mhCACI specifically considered populations in new, low-resource settings (Sangraula et al., 2021). Other contextual considerations included the setting, such as the acceptability and accessibility for a new population to attend an intervention in a church hall compared with a community centre (Chu & Leino, 2017). Intervention format could also be changed, for example, from a group to an individual program to accommodate community stigma around the illness or the treatment (Chu & Leino, 2017). Personnel involved in intervention delivery could also be adapted, for example including other stakeholders (Chu & Leino, 2017). Alternatively, resources may vary in the new context affecting availability of personnel; the mhCACI incorporated the need to utilise non-specialist workforce to deliver specialist interventions (Sangraula et al., 2021).

#### Fidelity

Eight of the frameworks considered fidelity to the original intervention or the role of core intervention components as part of the cultural adaptation (Barrera & Castro, 2006; Bernal et al., 1995; Chu & Leino, 2017; Heim & Kohrt, 2019; Marinez-Lora & Atkins, 2012; Sangraula et al., 2021; Sorenson & Harrell, 2021; Whitbeck, 2006). Two approaches to maintaining fidelity were identified. One approach suggested fidelity is maintained by identifying and then retaining the “core components” of an intervention during adaptation, as articulated by the ‘Cultural Treatment Adaptation Framework (CTAF)’ (Chu & Leino, 2017), the ‘4-Domain Cultural Adaptation Model (CAM4)’ (Sorenson & Harrell, 2021) and the mhCACI (Sangraula et al., 2021). Core components are generally considered the key active intervention components, the mechanisms that bring about the desired change (Wholey, 1987). However, the CTAF names the deep structural components of an intervention the “core components” and suggests these alone are the key active components.

The second approach suggested that, following identification of the key components, these may then need to be purposefully adapted to align with cultural views of the new population or address specific risk factors to achieve similar intervention outcomes. This approach was reflected in the ‘Ecological Validity Framework (EVF)’ (Bernal et al., 1995), the ‘Theoretical model for developing culturally specific preventions with Native American people’ (Whitbeck, 2006), Barrera and Castro’s (2006) ‘Heuristic’ framework, and the ‘New framework for cultural adaptation’ (Heim & Kohrt, 2019). In summary, the frameworks differed in their definitions of what constitutes a ‘key active component’ of an intervention and offered little guidance on how to identify these. They also differed in whether fidelity to the original intervention is achieved by retaining core intervention components or by retaining therapeutic outcomes by strategic adaptation, including of core components.

#### Engagement

Engagement was included within nine frameworks (Barrera & Castro, 2006; Bernal et al., 1995; Chu & Leino, 2017; Heim & Kohrt, 2019; Hwang, 2006; Leong & Lee, 2006; Naeem et al., 2016; Patchell et al., 2012; Sorenson & Harrell, 2021). The frameworks suggest strategic modification to promote engagement by identifying and addressing barriers, such as stigma (Barrera & Castro, 2006; Chu & Leino, 2017; Leong & Lee, 2006; Naeem et al., 2016; Sorenson & Harrell, 2021), and facilitators, such as orientation to the intervention to facilitate access (Hwang, 2006; Naeem et al., 2016; Sorenson & Harrell, 2021). Engagement is also enabled via the therapeutic relationship (Bernal et al., 1995; Chu & Leino, 2017; Hwang, 2006; Naeem et al., 2016; Patchell et al., 2012).

#### Cultural competence

Cultural competence was included within six frameworks (Hwang, 2006; Leong & Lee, 2006; Naeem et al., 2016; Patchell et al., 2012; Sorenson & Harrell, 2021; Whitbeck, 2006). Cultural competence of the practitioner is described as an important consideration for cultural adaptation in half the frameworks (Hwang, 2006; Leong & Lee, 2006; Naeem et al., 2016; Patchell et al., 2012; Sorenson & Harrell, 2021; Whitbeck, 2006); this encompasses the need for cultural knowledge and respect, cultural humility, culturally acceptable therapeutic approaches, and individualised therapeutic accommodation.

## Discussion

This systematic review identified 12 content-specific, cultural adaptation frameworks. Five main cultural adaptation elements were identified through the synthesis and there were commonalities in how the frameworks included these - all suggested that content modification was important for cultural adaptation, and context modification, intervention fidelity, engagement and cultural competence were also included in most frameworks. However, there was heterogeneity across the frameworks in terms of their application, specifically their intended usage, as well as how they defined and emphasised the different cultural adaptation elements. The interpretation of cultural competence in the frameworks varied, as did how the relationship between fidelity and key intervention components was guided by the frameworks during adaptation.

Dementia practitioners and researchers undertaking cultural adaptation can use a content-specific framework to support them to identify which of areas of the intervention can be targeted and enhance the transparency, consistency, and replicability of their adaption. However, the included frameworks were not designed for holistic adaptation of all types of psychosocial interventions for all populations, therefore when selecting a cultural adaptation framework, researchers and practitioners should consider the values and beliefs of the new population, the specific dementia intervention, and the purpose of the adaptation.

### Limitations of current frameworks

#### Maintaining fidelity is complex

The identification and retention of the key intervention components are key steps in any health intervention adaptation to ensure fidelity (Escoffery et al., 2019; Movsisyan et al., 2019), however this can be challenging. Firstly, the frameworks rely on adapters to identify the key components of an intervention; however, original intervention developers rarely articulate which components are key, and which are peripheral (Berkel et al., 2010). Dementia interventions are complex psychosocial interventions, and it can be difficult to define the specific mechanisms that ensure intervention success (Cuijpers et al., 2019; Lemmens et al., 2016; Teahan et al., 2020). For example, in a recent cultural adaptation of a caregiver intervention for people with dementia using the Cultural Treatment Adaptation Framework (CTAF) (Chu & Leino, 2017), one-to-one delivery was described as a key intervention component, differing from the original CTAF model where delivery mode is classified as a modifiable peripheral treatment component (Webster et al., 2023). To retain fidelity during cultural adaptation, intervention developers should clearly articulate the program theory, in particular they should describe the key active mechanism components (O’Cathain et al., 2019).

Secondly, retention of the key intervention components can be difficult if they are not culturally appropriate (Barrera & Castro, 2006; Lau, 2006; Resnicow et al., 1999). For example, a key component of a dementia intervention may be training for caregivers to improve their assertiveness when interacting with medical personnel (e.g., Gallagher-Thompson et al., 2003). Yet, for some cultures assertiveness is considered a disrespectful communication style, therefore that component would need to be considered for adaptation (Leong & Lee, 2006). One approach might be to increase the new population’s acceptance of the intervention’s key components during the adaptation process, suggested in the mhCACI framework (Sangraula et al., 2021). An alternative is to consider “functional fidelity”, whether the adapted intervention has the same outcome as the original (Evans et al., 2021). With this approach the actual intervention component is less important than the function it performs in achieving the desired program outcomes (Hawe, 2015). Using the previous example, the outcome is the caregiver’s ability to be an advocate, and the intervention could be adapted to include another approach in the place of assertiveness training to achieve this.

There was no consensus amongst frameworks on what elements of an intervention can be changed, how they can be changed and by how much; all key to achieving fidelity during cultural adaptation (Bernal et al., 2009). Nor was guidance provided on how much an intervention can be changed and still be considered an ‘adapted’ intervention, rather than a ‘new’ intervention. As such, dementia researchers and practitioners should be aware that cultural adaptation frameworks can provide guidance and offer consistency but do not solve the “Fidelity-Adaptation Dilemma”.

#### Traditional practices only sometimes considered

Most psychosocial interventions are based on Western values and understanding of health (Henrich et al., 2010). Most adaptation frameworks were developed in Western countries for the adaptation of these Western interventions for minority cultural groups or non-Western countries. It is important to consider that cultural perceptions of illness and healing may not align with the therapeutic foundations of an intervention (Castro et al., 2010; Falicov, 2009; Hall, 2001). Therefore, it is imperative that incorporation of traditional knowledge and local beliefs and values are considered within the adaptation process (Summerfield, 2013). Ownership of an adapted intervention can be facilitated by acknowledging historical trauma and power imbalances (Huria et al., 2019). The two included frameworks that focus specifically on Native Americans (Patchell et al., 2012; Whitbeck, 2006) offer thoughtful guidance to ensure culturally safe, responsible and respectful adaptations for Indigenous communities. Approaches that are sensitive to the needs of Indigenous communities can be relevant to other populations (Wilson et al., 2020), therefore these frameworks could be applied more broadly.

#### Ensuring cultural competence of practitioners is part of cultural adaptation

Elements of practitioner cultural competence were included in half the frameworks in this review. Effective delivery and outcomes of an adapted intervention may be compromised without a culturally competent practitioner working within a supportive structure. The inclusion of cultural competence recognises the importance of cross-cultural interactions and requires a practitioner to reflect on the impact of cultural knowledge, cultural differences, and how they will meet culturally unique needs (Cross et al., 1989). Program recipients’ perception of the cultural competence of their practitioner is positively correlated with intervention outcomes (Soto et al., 2018). However, there is a difference between a practitioner’s general competence and their cultural competence (Hayes et al., 2016; Imel et al., 2011). Although the inclusion of bilingual or bicultural workers may increase feasibility for diverse populations (Falicov, 2009), in effective cultural adaptation, workers, including bicultural workers, also receive cultural competence training (e.g., Belle et al., 2006; Cardemil et al., 2010; Wood et al., 2008). Further, a structure is required to support the incorporation of cultural competence into practice (Hwang, 2006).

### Implications for framework use

**Figure 2.**
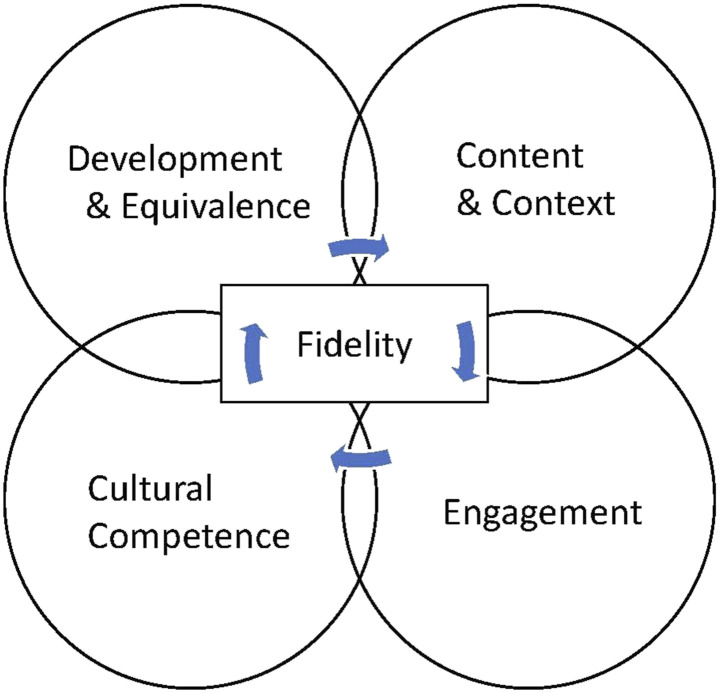
CAM4 adapted (to include fidelity throughout the cultural adaptation process). *Note.* Adapted from Sorenson and Harrell's (2021) 4-Domain Cultural Adaptation Model (CAM4).

This review found the CAM4 (Sorenson & Harrell, 2021) was the only framework that includes all the main synthesised adaptation elements: content, context, cultural competence, fidelity and engagement. However, the CAM4 incorporates fidelity only within the ‘Development and Equivalence’ phase of the framework. This means adapters identify and retain the key active mechanisms of the intervention at the start of the cultural adaptation process (Sorenson & Harrell, 2021). This review has highlighted the lack of consensus around how to maintain fidelity during cultural adaptation of complex psychosocial interventions, in particular the complexity surrounding the identification and retention of key components. Therefore, the CAM4 would be enhanced by considering fidelity not only at the outset but throughout each stage of the adaptation process. This concept is reflected in Figure 2.

### Strengths and limitations of this systematic review

This review contributes to cultural adaptation literature by building on recent reviews of adaptation frameworks and guidelines (Escoffery et al., 2019; Movsisyan et al., 2019). By focussing on the content perspective of cultural adaptation, an area included in process frameworks with little guidance or detail as to *what* to do, this synthesis of 12 frameworks collates important available information to support dementia researchers and practitioners in the cultural adaptation process.

This review has several limitations. First, our search terms were in English and most identified frameworks were developed in the United States, therefore caution should be taken when interpreting the results in a global context. Second, no quality appraisal or risk of bias evaluation was conducted for the review as the purpose was to identify available frameworks rather than appraise the data. To increase the rigor of this review, two reviewers were used throughout the screening and data extraction phases. The process of data extraction and synthesis of theoretical constructs was reviewed by all authors to ensure accuracy. An additional limitation is that cultural adaptation is guided by conceptual and theoretical papers as much as practical frameworks and these were excluded from the review. However, the included frameworks referenced many of the leading theoretical references to support their framework development or articulate their definitions and as such the concepts form part of the review. Finally, we included only original papers describing a framework therefore may have omitted elaborations or clarifications included in subsequent publications (e.g., Naeem et al., 2019; Rathod et al., 2019).

## Conclusion

Comprehensive cultural adaptation frameworks are available to support cultural adaptation of psychosocial dementia interventions. These were predominantly developed for minority cultural groups in the USA, for adaptation of psychology interventions. This systematic review provides a summary of the main elements of content-specific cultural adaptation frameworks and a synthesis of how these can be applied during the adaptation process. Adapters should follow the steps for adaption (i.e., planning, conducting, testing), apply the selected content-specific framework to guide the adaptation, and draw on the extensive cultural adaptation literature to achieve meaningful and authentic cultural adaptation of dementia interventions. Consideration should be given to the impact of cultural competence. Cultural adaptation frameworks would be enhanced by incorporating consideration of fidelity throughout the adaptation process. To further ensure fidelity during adaptation, more work is required by dementia researchers and program developers to both articulate the key intervention components and ensure practitioners understand how they work.

## Supplemental Material

Supplemental Material - Frameworks for cultural adaptation of psychosocial interventions: A systematic review with narrative synthesis Click here for additional data file. Supplemental Material for Frameworks for cultural adaptation of psychosocial interventions: A systematic review with narrative synthesis by Sally Day, Kate Laver, Yun-Hee Jeon, Kylie Radford and Lee-Fay Low in Dementia

## Appendix

**Table 1.** Databases searched.

Database	Coverage
Ovid	
MEDLINE(R) ALL	1946 to Present
Embase Classic + Embase	1947 to Present
PsycINFO	1806 to Present
Elsevier	
Scopus	1960 to Present
EBSCOhost	
CINAHL Complete	1937 to Present

**Table 2.** MEDLINE search strategy.

1	Cognitive Behavioral Therapy/ (29778)
2	“cognitive behavio?r* therap*".mp. (38768)
3	cogniti* rehabilitation.mp. (2258)
4	“cognitive stimulation therap*".mp. (157)
5	Occupational Therapy/ (14680)
6	“occupational therap*".mp. (23289)
7	Psychosocial Intervention/ (817)
8	“psychosocial intervention*”.mp. (7709)
9	psychoeducation*.mp. (6750)
10	“skills training".mp. (8209)
11	"psycholog* intervention*".mp. (8585)
12	(psychosocial adj2 (training or educati* or treatment* or support* or program* or intervent* or care*)).tw. (18523)
13	psychotherap*.mp. (98943)
14	"behavio?r* intervention*".mp. (1303)
15	"social intervention*".mp. (1260)
16	"function* intervention*".mp. (348)
17	(behavio?r* adj2 (training or educati* or treatment* or support* or program* or intervent* or care*)).tw. (56172)
18	(function* adj2 (training or educati* or program* or intervent* or care*)).tw. (13572)
19	(social adj2 (training or educati* or treatment* or support* or program* or intervent* or care*)).tw. (82885)
20	cross?cultur*.mp. (182)
21	cultur* adapt*.mp. (9364)
22	cultural* tailor*.mp. (1709)
23	cultural* safe*.mp. (1123)
24	cultural* responsiv*.mp. (917)
25	cultur* adjust*.mp. (195)
26	cultur* modif*.mp. (280)
27	frame?work*.mp. (383614)
28	model*.tw. (3613489)
29	theor*.tw. (785794)
30	exp Guideline/ (37531)
31	guideline*.tw. (453373)
32	20 or 21 or 22 or 23 or 24 or 25 or 26 (13542)
33	27 or 28 or 29 or 30 or 31 (4787045)
34	1 or 2 or 3 or 4 or 5 or 6 or 7 or 8 or 9 or 10 or 11 or 12 or 13 or 14 or 15 or 16 or 17 or 18 or 19 (318938)
35	32 and 34 (1319)
36	33 and 35 (521)
